# Fish RIP1 Mediates Innate Antiviral Immune Responses Induced by SGIV and RGNNV Infection

**DOI:** 10.3389/fimmu.2020.01718

**Published:** 2020-08-04

**Authors:** Xin Zhang, Zetian Liu, Siting Wu, Mengshi Sun, Jingguang Wei, Qiwei Qin

**Affiliations:** ^1^Joint Laboratory of Guangdong Province and Hong Kong Region on Marine Bioresource Conservation and Exploitation, College of Marine Sciences, South China Agricultural University, Guangzhou, China; ^2^Guangdong Laboratory for Lingnan Modern Agriculture, Guangzhou, China; ^3^Laboratory for Marine Biology and Biotechnology, Qingdao National Laboratory for Marine Science and Technology, Qingdao, China

**Keywords:** grouper, RIP1, SGIV, RGNNV, interaction

## Abstract

Receptor interacting protein 1 (RIP1) is an essential sensor of cellular stress, which may respond to apoptosis or cell survival and participate in antiviral pathways. To investigate the roles of fish RIP1 in Singapore grouper iridovirus (SGIV) and red-spotted grouper nervous necrosis virus (RGNNV) infection, a RIP1 homolog from orange-spotted grouper (*Epinephelus coioides*) (EcRIP1) was cloned and characterized. EcRIP1 encoded a 679 amino acid protein that shares 83.28% identity with that of *Perca flavescens* and contained a homologous N-terminal kinase (S-TKc) domain, a RIP isotype interaction motif (RHIM), and a C-terminal domain (DD). EcRIP1 was predominantly detected in immune tissues, and its expression was induced by RGNNV or SGIV infection *in vitro*. Subcellular localization showed that EcRIP1 was distributed in the cytoplasm with point-like uniform and dot-like aggregation forms. Overexpression of EcRIP1 inhibited SGIV and RGNNV replication and positively regulated the expression levels of interferon (IFN) and IFN-stimulated genes and pro-inflammatory factors. EcRIP1 may interact with grouper tumor necrosis factor receptor type 1-associated DEATH domain protein (EcTRADD) to promote SGIV-induced apoptosis, and interact with grouper Toll/interleukin-1 receptor (TIR) domain containing adapter inducing interferon-β (EcTRIF) and participate in Myeloid Differentiation Factor 88 (MyD88)-independent toll-like receptor (TLR) signaling. EcRIP1 may also interact with grouper tumor necrosis factor receptor-associated factors (TRAFs) as intracellular linker proteins and mediate the signaling of various downstream signaling pathways, including NF-κB and IFN. These results suggest that EcRIP1 may inhibit SGIV and RGNNV infection by regulating apoptosis and various signaling molecules. Our study offers new insights into the regulatory mechanism of RIP1-related signaling, and provides a novel perspective on fish diseases mediated by RIP1.

## Introduction

Cells can respond to the stress of distinct pathogens (e.g., viruses and bacteria) by regulating cell signaling pathways mediated by nuclear factor-κB (NF-κB), interferon (IFN), or p53 transcription factors (1, 2). These cellular signaling pathways can be induced by target genes to regulate a variety of vital biological processes, including immune responses, inflammatory reactions, and apoptosis (3). For example, activation of NF-κB has the dual effects of anti-apoptosis or pro-apoptosis (2). In an attempt to understand how cells balance and regulate survival and death decisions under external stimuli, we began to focus on receptor-interacting protein (RIP) family kinases, which are thought to be essential sensors of cellular stress (4, 5).

In humans, the RIP serine/threonine kinase family contains seven members that share a homologous N-terminal kinase domain but have unique recruitment domains (4). RIP1 contains an N-terminal kinase domain, an intermediate domain (ID), and a C-terminal death domain (DD) (1, 6). The DD of RIP1 can bind to the death receptor, and it may be related to the adapter proteins that also contain a DD, such as TNFR-related DD (TRADD) and Fas-related DD (FADD) (1, 4). The ID of RIP1 contains a RIP homotypic interaction motif (RHIM) that interacts with other proteins containing this motif, such as RIP3 and TIR domain-containing adapter inducing interferon-β (TRIF, also known as TICAM1), which can induce NF-κB activation (7, 8). Additionally, the kinase domain of RPI1 may play an important role in cell necrosis (1).

The complex structure of RIP1 means that it may participate in antiviral pathways and respond to multiple cellular signals, such as tumor necrosis factor (TNF)-, Toll-like receptor (TLR), and retinoic acid-inducible gene-I (RIG-I)-like helicase (RLH) ligand-associated signal transduction (9). RIP1 can recruit TRADD through the carboxy-terminal DD, while the amino terminal domain of TRADD (TRADD-N) can recruit the TNF receptor-associated factor (TRAF) 2 adaptor protein. Furthermore, complexes of them can regulate the TNF-R1-mediated apoptosis pathway (9, 10).

Six mammalian TRAFs (TRAF1–6) have been identified that can activate NF-κB and activator protein-1 family transcription factors to participate in cellular proliferation, differentiation, and regulation of the immune response (10). Recent studies have shown that RIP1 may physically interact with TRIF through an ID, cooperating with DD to induce apoptosis. Together with TRAF6, they are recruited to the TRIF-dependent TLR3/4 signaling involved in type I IFN-β and contribute to TRIF-induced NF-κB activation (11). Furthermore, studies have shown that RIP1 is involved in the antiviral pathway of RIG-like receptor signaling. RIP1 forms a complex with the E3 ubiquitin ligase TRAF2 and with FADD and TRADD, triggering activation of NF-κB and IRF3, which collaborate to induce an antiviral type I IFN response (12).

Previous experiments have shown that apoptosis induced by RIP1 in combination with DD-containing proteins or TRIF during viral infection represents an important host defense mechanism that can limit the spread of infection. RIP1 is also essential for TLR3/TRIF-dependent signaling targeting viral RNA and RLH-Cardiff-dependent antiviral immune responses, such as controlling human inflammation and anti-viral responses in the ocular surface and inhibiting vesicular stomatitis virus replication (13, 14).

The grouper (*Epinephelus* spp.) is an economically important fish farmed in southern China and Southeast Asia. However, frequent outbreaks of viral diseases in recent years have caused heavy economic losses in the grouper industry. The most typical pathogens are Singapore grouper iridovirus (SGIV) and red grouper neuronecrosis virus (RGNNV) (15, 16). SGIV is an enveloped double-stranded large DNA virus with a genome of 140,131 base pairs (bp) that encode 162 open reading frames (ORFs) (17). As described previously, the typical apoptosis in fathead minnow (FHM) epithelial cells induced by SGIV can be regulated by activation of the c-Jun N-terminal kinase and NF-κB pathways (18, 19). RGNNV is a non-enveloped icosahedral RNA virus whose genome consists of two single-stranded positive-sense RNAs (20); RNA1 (3.1 kb) encodes the RNA-dependent RNA-polymerase (RdRp), and RNA2 (1.4 kb) encodes the capsid protein (CP) (21). To study the prevention and treatment of grouper virus diseases, many immune genes have been cloned with a focus on the anti-virus immune network (22–24). However, research on the function of the RIP1 gene is rare and has mainly focused on humans and amphibians (1, 8, 9). The roles of RIP1 and its interaction proteins in the replication of SGIV or RGNNV have not been reported previously.

In this study, a key apoptosis-related gene (RIP1) from orange-spotted grouper (*Epinephelus coioides*) (EcRIP1) was cloned and identified. We investigated the antiviral effects of EcRIP1 during SGIV and RGNNV infection and evaluated its interaction with key proteins in multiple signaling pathways. Our results will provide new insights into the function of fish RIP1 in virus infection.

## Materials and Methods

### Fish, Cells and Viruses

Juvenile orange-spotted groupers (weight 30–40 g) used in this study were purchased from Wenchang Marine Fish Farm, Hainan Province, China. They were kept in a laboratory recirculating seawater system at 24–28°C and fed twice daily for 2 weeks.

Grouper spleen (GS) (25) and FHM epithelial (26) cell lines were grown at 28°C in Leibovitz's L15 and M199 culture media, respectively, with 10% fetal bovine serum (Gibco, USA). The virus stocks of SGIV and purified SGIV were propagated in GS cells, whereas the RGNNV stocks were propagated in grouper brain (GB) cells; the titers of the viruses were determined in GS cells and GB cells, respectively, that were both grown in Leibovitz's L15 medium containing 10% fetal bovine serum (27, 28). Virus stocks were maintained at −80°C.

### Cloning of EcRIP1 and Bioinformatic Analysis

Based on the expressed sequence tag (EST) sequences of EcRIP1 from the grouper spleen transcriptome (29), primers (Table 1) were designed, and the full-length EcRIP1 as well as its domain sequence were cloned by polymerase chain reaction (PCR) amplification. The sequence of EcRIP1 was analyzed using the BLAST program (http://www.ncbi.nlm.nih.gov/blast), and the conserved domains or motifs were predicted using the Conserved Domains program (https://www.ncbi.nlm.nih.gov/cdd/). Amino acid alignments were carried out using Clustal X1.83 software and edited using the GeneDoc program. The phylogenetic analysis was conducted using the neighbor-joining (NJ) method in MEGA 6.0 software.

**Table 1:** Primers used in this study.

**Name**	**Sequence(5′-3′)**
EcRIP1-ORF-F	ATGGCCACCGCGCCACAGCCTTCAC
EcRIP1-ORF-R	CTAGGAAGAAGAACCGCAGGCGTTC
C1-EcRIP1-F	GATCTCGAGCTATGGCCACCGCGCCACAGCCTTCAC
C1-EcRIP1-R	CAGAATTCCTAGGAAGAAGAACCGCAGGCGTTC
RFP-EcRIP1-F	GCGAATTCATGGCCACCGCGCCACAGCCTTCAC
RFP-EcRIP1-R	GCCTCGAGCTAGGAAGAAGAACCGCAGGCGTTC
HA-EcRIP1-F	GCGAATTCATGGCCACCGCGCCACAGCCTTCAC
HA-EcRIP1-R	GCCTCGAGCTAGGAAGAAGAACCGCAGGCGTTC
EcRIP1-RT-F	TGTGGTTTGGGTCATCCT
EcRIP1-RT-R	TTGGCCGTTGTATTGGA
C1-EcRIP1-S_TKc-F	GATCTCGAGCTATGCTCATCAAAAAGGAGGCCC
C1-EcRIP1-S_TKc-R	CAGAATTCCTAGAAGAAGTCGTAGCCCTCT
C1-EcRIP1-DD-F	GATCTCGAGCTATGGGCATACTGAAATACGAGG
C1-EcRIP1-DD-R	CAGAATTCCCTAGCAGGCGTTCAGTATTTTCTGG
C1-EcRIP1-ΔS_TKc-F	GATCTCGAGCTATGTTCCCTTTCTACACTGAAAAG
C1-EcRIP1-ΔS_TKc-R	CAGAATTCCCTAGGAAGAAGAACCGCAGGC
C1-EcRIP1-ΔDD-F	GATCTCGAGCTATGGCCACCGCGCCACAGCCTTCAC
C1-EcRIP1-ΔDD-R	CAGAATTCCCTATTCCTTGATGGGAGAGTTAAC
siRNA1	CAGGUGGUGUUGAAGACCAUGUACA
siRNA2	GCUACGACUUCUUCUUCCCUUUCUA
siRNA3	CAGCAACAUCCACAUUCCCACUAUG
RGNNV CP-RT-F	CAACTGACAACGATCACACCTTC
RGNNV CP-RT-R	CAATCGAACACTCCAGCGACA
RGNNV RdRp-RT-F	GTGTCCGGAGAGGTTAAGGATG
RGNNV RdRp-RT-R	CTTGAATTGATCAACGGTGAACA
SGIV MCP-RT-F	GCACGCTTCTCTCACCTTCA
SGIV MCP-RT-R	AACGGCAACGGGAGCACTA
SGIV VP19-RT-F	TCCAAGGGAGAAACTGTAAG
SGIV VP19-RT-R	GGGGTAAGCGTGAAGAC
ICP-18-RT-F	ATCGGATCTACGTGGTTGG
ICP-18-RT-R	CCGTCGTCGGTGTCTATTC
Actin-RT-F	TACGAGCTGCCTGACGGACA
Actin-RT-R	GGCTGTGATCTCCTTCTGCA
EcIL-1β-RT-F	AACCTCATCATCGCCACACA
EcIL-1β-RT-R	AGTTGCCTCACAACCGAACAC
EcTNFα-RT-F	GTGTCCTGCTGTTTGCTTGGTA
EcTNFα-RT-R	CAGTGTCCGACTTGATTAGTGCTT
EcIL-8-RT-F	GCCGTCAGTGAAGGGAGTCTAG
EcIL-8-RT-R	ATCGCAGTGGGAGTTTGCA
EcIRF3-RT-F	GACAACAAGAACGACCCTGCTAA
EcIRF3-RT-R	GGGAGTCCGCTTGAAGATAGACA
EcIRF7-RT-F	CAACACCGGATACAACCAAG
EcIRF7-RT-R	GTTCTCAACTGCTACATAGGG
EcISG15-RT-F	CCTATGACATCAAAGCTGACGAGAC
EcISG15-RT-R	GTGCTGTTGGCAGTGACGTTGTAGT
EcMyD88-RT-F	AGCTGGAGCAGACGGAGTG
EcMyD88-RT-R	GAGGCTGAGAGCAAACTTGGTC
EcTRAF6-RT-F	CCCTATCTGCCTTATGGCTTTGA
EcTRAF6-RT-R	ACAGCGGACAGTTAGCGAGAGTAT
EcMDA5-RT-F	ACCTGGCTCTCAGAATTACGAACA
EcMDA5-RT-R	TCTGCTCCTGGTGGTATTCGTTC
EcLGP2-RT-F	TGGTGGTACGCTATGGACTGC
EcLGP2-RT-R	TTGTAGCTCAGTTATCTTTGTGCGA
EcISG56-RT-F	CTGTTGTTACGCACGGAGGAT
EcISG56-RT-R	CCTGCGTGGGTTCATTCAGT
EcIFN2-RT-S	TACAGCCAGGCGTCCAAAGCATC
EcIFN2-RT-R	CAGTACAGGAGCGAAGGCCGACA

### RNA Isolation and Quantitative Real-Time (qRT)-PCR

Total RNA was extracted using SV Total RNA Isolation Kit (Promega, USA) according to the manufacturer's protocol. The quality of total RNA was assessed by electrophoresis on 1% agarose gel. Total RNA was reverse transcribed to synthesize the first-strand cDNA using the ReverTra Ace kit (Toyobo, Japan) according to the manufacturer's instructions.

qRT-PCR was performed in an Applied Biosystems Quant Studio 3 Real Time Detection System (Thermofisher, USA) to check the transcriptional expression level of host immune genes and virus genes. Each assay was carried out in triplicate with the following cycling conditions: 1 min for activation at 95°C followed by 40 cycles for 15 s at 95°C, 15 s at 60°C, and 45 s at 72°C. Table 1 lists the primers used. The expression levels of target genes were normalized to β-actin and calculated using the 2^−ΔΔ*CT*^ method. The data are represented as mean ± standard deviation (SD).

### Expression Patterns for EcRIP1 in Grouper

To elucidate the tissue distribution of EcRIP1 in healthy orange-spotted grouper, samples of head kidney, liver, spleen, kidney, brain, intestine, skin, muscle, heart, and blood from six fish were collected for RNA extraction and further qRT-PCR analysis. To detect the expression profiles of EcRIP1 in response to virus infection, additional orange-spotted groupers were infected with SGIV or RGNNV and harvested at 0, 3, 18, 24, 30, and 42 h post-infection (h.p.i.). Total RNA was extracted and the expression of EcRIP1 was analyzed using qRT-PCR.

### Plasmid Construction and Cell Transfection

To study subcellular localization and co-localization of interacting proteins, the full-length EcRIP1 and its four domains (S_Tkc, DD, ΔS_Tkc, and ΔDD) and the full-length EcTRIF, EcMyD88, EcTRAFs, EcTRADD, EcFADD were inserted into the pEGFP-C1 vector, respectively. In addition, pcDNA3.1-3HA-RFP-EcRIP1 red fluorescent vector was constructed for co-localization. For protein function and co-immunoprecipitation tests, they were all inserted into the pcDNA3.1-3HA vector. All recombinant plasmids were confirmed by DNA sequencing. Cell transfection was carried out using Lipofectamine 2000 transfection reagent (Invitrogen) as described previously (27). Briefly, FHM and GS cells were seeded in 24-well cell culture plates or 6-well plates at 60–70% confluence. They then were incubated with the mixture of Lipofectamine 2000 and plasmids for 6 h, after which the mixture was replaced with fresh normal medium.

### Confocal Laser Scanning Microscope and Fluorescence Microscopy

GS cells seeded on the cell culture dish (10 cm × 10 cm) were transfected with a total of 400 ng of a given expression plasmid. At 48 h after transfection, the cells were washed with phosphate buffered saline (PBS), fixed with 4% polyformaldehyde for 1 h at 4°C, and then stained with Mitotracker red CMXRos and 4, 6-diamidine 2-phenylindole (DAPI). After washing with PBS, the cells were imaged under a confocal laser scanning microscope. In addition, we used a fluorescence microscope (Leica, Germany) to observe and photograph the morphology of the cells.

### Reporter Gene Assays

To evaluate the activity patterns of NF-κB and IFN promoted by EcRIP1 and its domain or other interacting proteins, luciferase plasmids including IFN-sensitive response elements IFN3-Luc and nuclear factor NF-κB (Clontech, USA) were used for co-transfection. Briefly, GS cells were transiently transfected with the luciferase plasmid along with the corresponding expression vectors using Lipofectamine 2000 reagent. PRL-SV40 *Renilla* luciferase vector was used as the internal control. The luciferase reporter gene assay system (Promega) was used to measure the luciferase activity of total cell lysates. A total of 50 ng of SV40 was included to normalize the luciferase activities. Cells were then harvested to measure the luciferase activities using the Dual-Luciferase® Reporter Assay System (Promega) at 48 h.p.i. according to the manufacturer's instructions.

### Small Interfering RNA (siRNA)-Mediated EcRIP1 Knockdown

To knockdown the expression level of EcRIP1 in GS cells, three siRNAs targeting different sequences of EcRIP1 mRNA were commercially synthesized by Invitrogen. GS cells were transfected with one of three siRNAs (Table 1) or the same volume of the negative control, and then they were infected with SGIV/RGNNV or left untreated. At the end of the corresponding incubation period, the total RNA of the extracted cells was detected by qPCR.

### Co-immunoprecipitation Assays and Western Blot

GS cells in the cell culture dish (10 cm × 10 cm) were transfected with 16 mg of DNA plasmid (8 mg/each expression vector) for 48 h. The transfected GS cells were lysed in radio-immuno-protein assay buffer containing 100 mM NaCl, 0.5% NP-40, 1 mM EDTA, and 20 mM Tris (pH 8.0). The Dynabeads™ Protein G Immunoprecipitation Kit (Invitrogen) was used to process collected cell samples. Proteins were separated by 12% SDS-PAGE and transferred onto Immobilon-P polyvinylidene difluoride membranes (Millipore, Temecula, CA, USA). Blots were incubated with the indicated primary antibody: anti-GFP (1:1000 dilution) or anti-3HA (1:1000 dilution). Subsequently, they were incubated with horseradish peroxidase (HRP)-conjugated anti-rabbit IgG antibody (1:5000 dilution) or HRP-conjugated anti-mouse IgG antibody (1:5000 dilution). Immunoreactive proteins were observed using an Enhanced HRP-DAB Chromogenic Substrate Kit (Tiangen, Beijing, China).

### Yeast Two-Hybrid Analysis

For the yeast two-hybrid analysis, the corresponding genes were cloned separately into pGBKT7 and pGADT7 vectors, and pGBKT7-BD as well as pGADT7-EcRIP1/EcTRIF/EcMyD88 plasmids were constructed for self-activation verification. Additionally, pGBKT7-RIP1 was constructed for interaction verification. All constructed plasmids were verified by sequencing. The two plasmids were co-transformed into *Saccharomyces cerevisiae* strain Y2H Gold. The transformants were tested on SD/-leu/-trp and SD/-leu/-trp/-his/-ade/X-α-gal/AbA media.

### Analysis of Cell Apoptosis

FHM cells overexpressing pcDNA3.1-3HA, pcDNA3.1-EcRIP1, pcDNA3.1- EcRIP1+EcTRADD, or pcDNA3.1-EcRIP1+EcFADD were infected with SGIV at multiplicity of infection of 2. Cells were stained with Hoechst 33342 at 24 h.p.i., and the morphologies of apoptotic corpuscles were observed under a fluorescence microscope. Additionally, other cells were harvested, washed twice with PBS, and resuspended in 1 × binding buffer at a concentration of 1 × 106 cells/ml. Next, 100 μl of the solution (1 × 10^5^ cells) were transferred to a 5 ml culture tube, and 5 μl of FITC Annexin V and 5 μl propidium iodide were added. The cells were gently vortexed and incubated for 15 min at 25°C in the dark. Four hundred microliters of 1 × binding buffer were added to each tube. The cells were analyzed by flow cytometry within 1 h. Data analysis was performed with FlowJo V10.

### Caspase Activation Assay

Caspase-3 activity was measured as previously described (30). The activities of caspase-3 were measured using a DEVD-AFC device (BioVision). Cells were collected from monolayers and then lysed in 60 μl of cell lysis buffer for 10 min on ice. After centrifugation, 45 μl of supernatant were extracted and added to a 96-well plate. Next, 50 μl of 2 × reaction buffer (containing 10 Mm dithiothreitol) were added to each sample. Finally, 5 μl of the 1 Mm DEVD-AFC substrate (50 μM final concentration) were added, and the mixture was incubated for 1–2 h at 37°C. The levels of cleaved caspase substrate were measured using a spectrofluorometer (Molecular Devices).

### Statistical Analysis

Statistical analyses (one-way analysis of variance) were performed using SPSS version 20. Differences were considered to be statistically significant at ^*^P < 0.05 and ^**^P < 0.01.

## Results

### Sequence Characterization of EcRIP1

**Figure 1:**
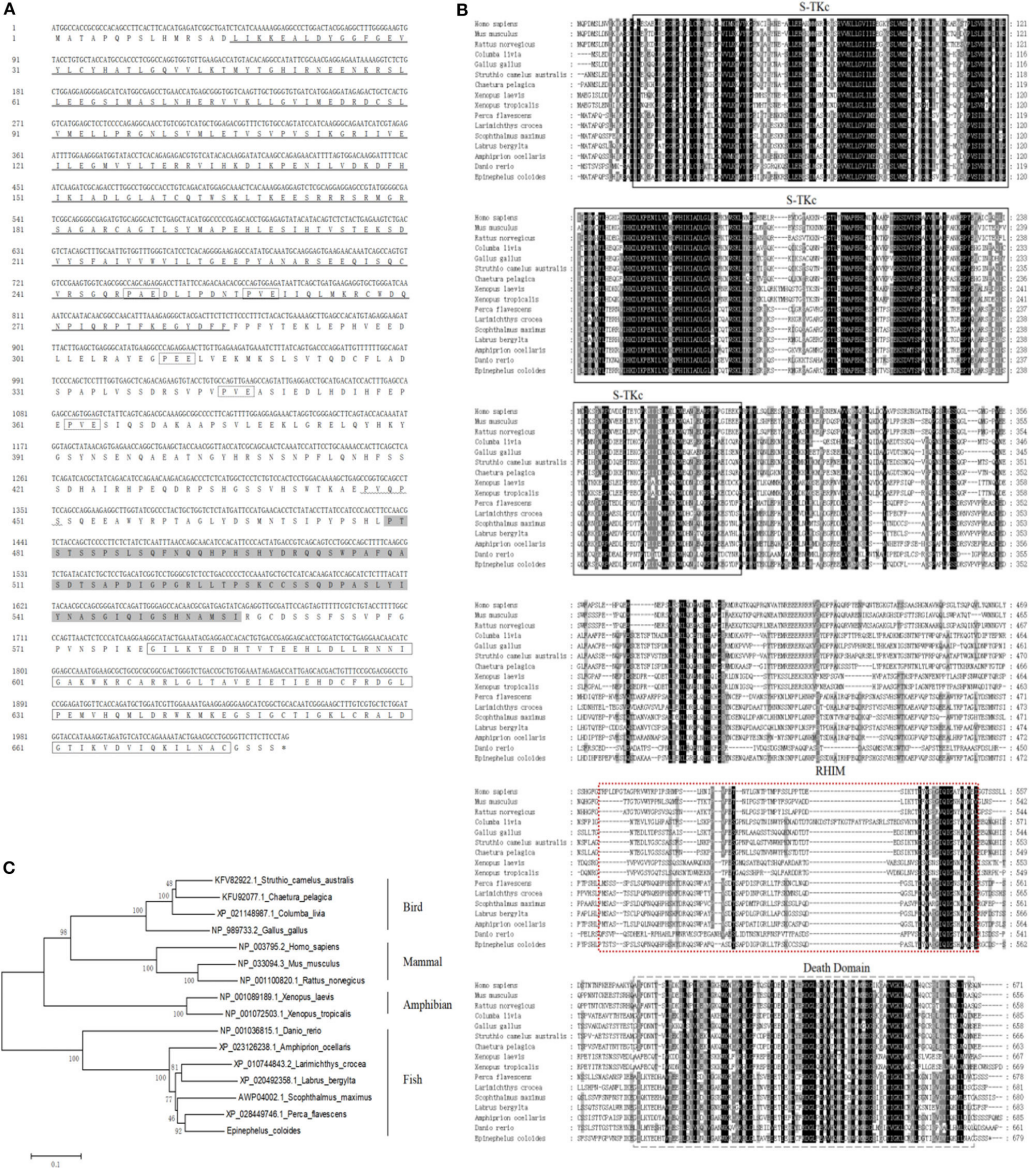
Molecular cloning of grouper RIP1. **(A)** Nucleotide sequence of EcRIP1 and the deduced amino acid sequence. The conserved domains S-TKc (at positions 16–285), RHIM (at positions 479–556), and DD (at positions 579–675) are underlined, shaded, and bordered. The underline of wavy line indicates the TRAF2/3 binding motif, and the dotted frame indicates the TRAF6 binding motif. **(B)** Multiple sequence alignments of RIP1s. The full-length amino acid sequences of RIP1s from typical organisms were aligned using the Clustal X 1.83 program (http://www.ebi.ac.uk/tools/clustalw2). **(C)** The phylogenetic tree was constructed according to the alignment of amino acid sequences using the NJ method within MEGA 4.0, with 1,000 bootstrap replications. The bootstrap values are indicated at the nodes of the tree. The GenBank accession number of each species is listed to the right of the species name.

Based on the EST sequences from the grouper spleen transcriptome, the full-length cDNA of EcRIP1 was amplified. It contains a 179 bp 5′-terminal untranslated region (UTR), a 1,042 bp 3′-UTR including a poly (A) tail, and a 2,040 bp ORF that encodes a putative 679 amino acid protein. Conserved Domains search analysis showed that three conserved domains—S-TKc at positions 16–285, RHIM at positions 479–556, and DD at positions 579–675—are present in EcRIP1. EcRIP1 contains conserved TRAF2/3 binding sites (PXQXT/S) and TRAF6 binding sites (PXEXX) (Figure 1A), suggesting its potential ability to recruit TRAFs. BLAST analysis revealed that EcRIP1 shares 83.28% identity with that of *Perca flavescens* (XP_028449746.1). Multiple sequence alignments were carried out using Clustal X multiple-alignment software, and a phylogenetic tree was constructed using the NJ method with 1,000 bootstraps. EcRIP1 is clustered in the Osteichthyes branch, and the RIP1 subfamily is conservative among Osteichthyes (Figures 1B,C).

### Tissue Distribution and Expression Profiles of EcRIP1 *in vivo*

**Figure 2:**
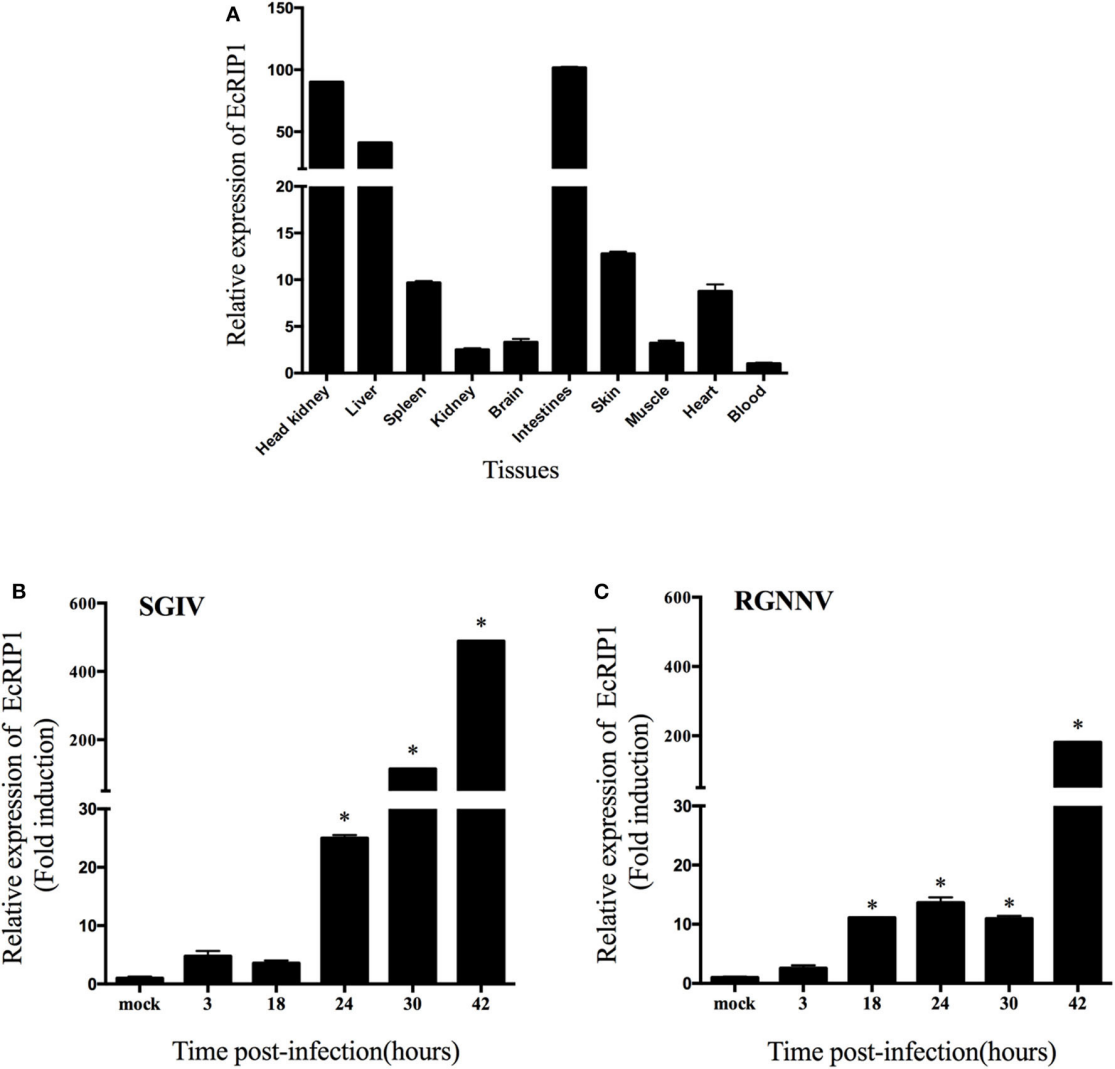
Expression patterns of EcRIP1. **(A)** Tissue distribution of EcRIP1 in healthy groupers and expression of EcRIP1 in the spleen of groupers at different time points post-SGIV **(B)** and RGNNV **(C)** infection. Error bars represent standard deviations of triplicate measurements, and *indicates that the means were significantly different at *P* < 0.05.

The transcript levels of EcRIP1 in different tissues from healthy juvenile orange-spotted groupers were analyzed using qRT-PCR. EcRIP1 was distributed in all examined tissues and was detected predominantly in head kidney, liver, spleen, intestines, and skin (Figure 2A). After SGIV and RGNNV infection, the transcription levels of EcRIP1 in challenged GS cells were both significantly up-regulated; the peak levels were reached at 42 h.p.i., with values that were 489- and 180-fold higher, respectively, than the level in the mock-infected cells (Figures 2B,C).

### Cellular Localization of EcRIP1 and Activation of NF-κB in GS Cells

**Figure 3:**
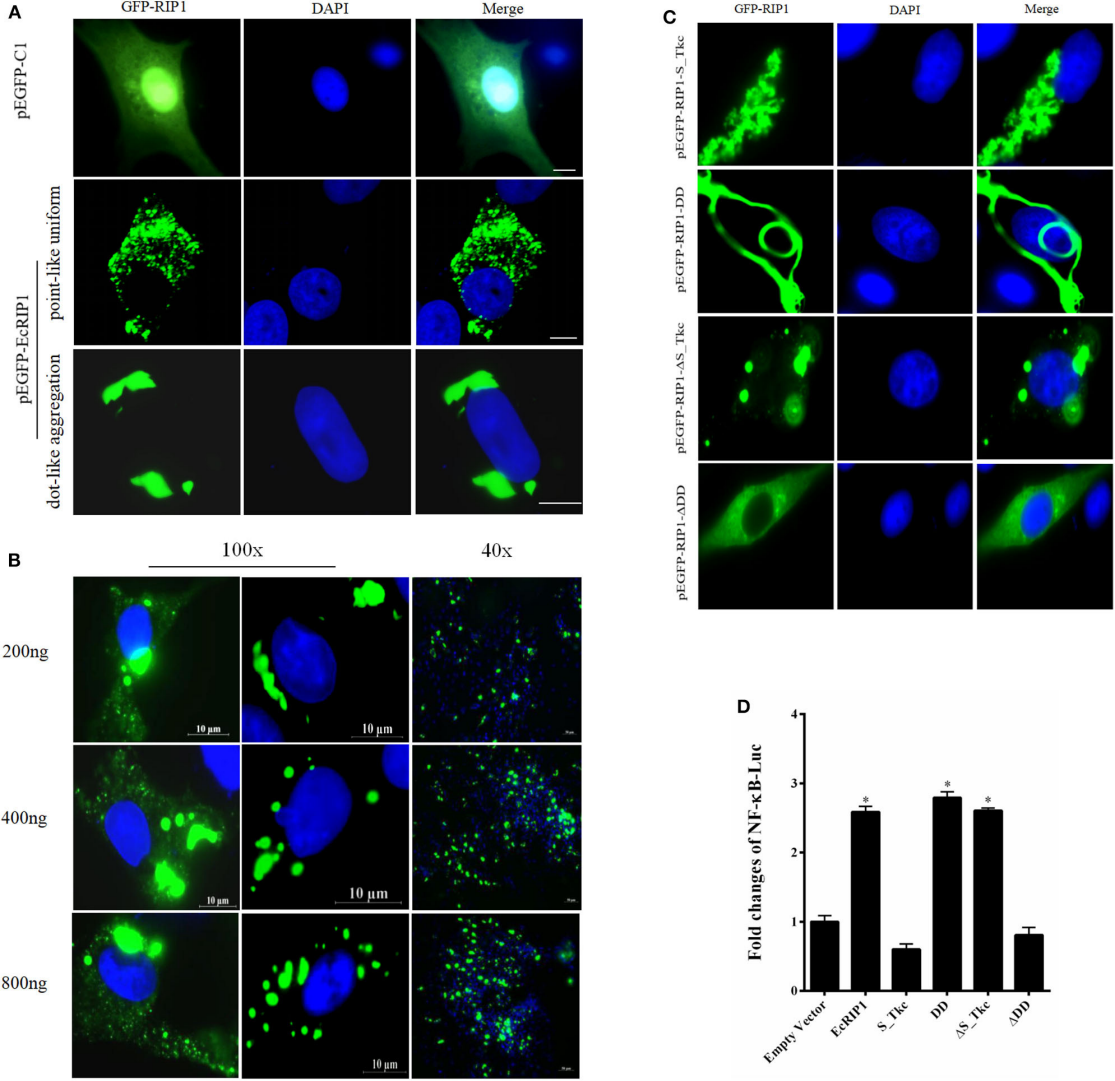
Cellular localization of EcRIP1. **(A,C)** GS cells were transfected with pEGFP-C1, pEGFP-EcRIP1, pEGFP-EcRIP1-S-TKc, pEGFP-EcRIP1-DD, pEGFP-EcRIP1-ΔS-TKc, and pEGFP-EcRIP1-ΔDD plasmids using Lipofectamine 2000. Cells were stained with DAPI at 24 h post-transfection and imaged under fluorescence microscopy. **(B)** EcRIP1 protein uniform in GS cells was verified by different doses of transfection, and the images were taken under 100x and 40x lenses, respectively. **(D)** Analysis of the effect of EcRIP1 and its truncated mutants on NF-κB activation. *P < 0.05.

The green fluorescence in cells transfected with pEGFP-C1 was distributed throughout the cytoplasm and nucleus. In cells transfected with pEGFP-EcRIP1, the green fluorescence was distributed in the cytoplasm in two forms: point-like uniform and dot-like aggregation forms (Figure 3A). Further, we verified the EcRIP1 protein uniform by transfection of different doses, and observed under different fields of view (Figure 3B). To investigate the function of EcRIP1, several truncated mutants were constructed and their cellular localization was observed (Figure 3C), and reporter assays were performed (Figure 3D). The S-TKc domain of EcRIP1was observed in the cytoplasm as an irregular cluster, whereas DD exhibited a clear pattern of discrete and interconnecting cytoplasmic filaments resembling death effector filaments (31). Green fluorescence appeared as a dot-like and filament-like diffuse distribution in the truncated mutants containing the ID together with the DD (ΔS-TKc). In contrast, ΔDD was observed in the cytoplasm (Figure 3C). The truncated mutants containing only the kinase domain of EcRIP1 did not activate the NF-κB response, whereas the truncated mutants containing the DD alone or together with the ID were more effective than the full-length EcRIP1 (Figure 3D).

### Antiviral Effects of EcRIP1 on Fish Virus Replication *in vitro*

**Figure 4:**
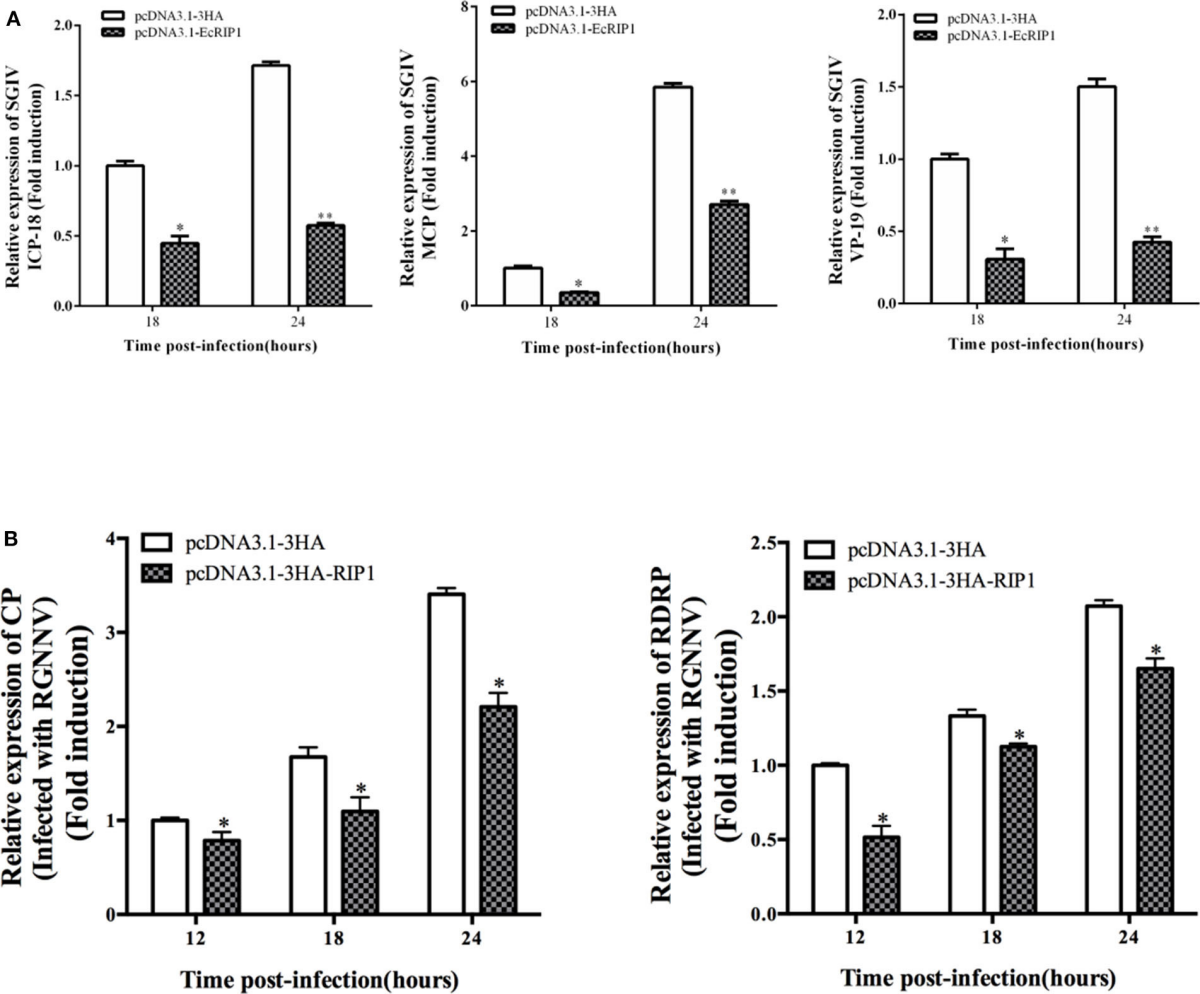
Activities of EcRIP1 during SGIV and RGNNV infection and replication *in vitro*. Viral gene transcription of SGIV **(A)** or RGNNV **(B)** in EcRIP1-overexpressing cells. EcRIP1-overexpressing cells were infected with RGNNV or SGIV and collected at 18 and 24 or 12, 18, and 24 h.p.i. to measure expression via qPCR of ICP-18, MCP, and VP19 of SGIV and CP and RdRp of RGNNV (*n* = 3, mean ± SD). *P < 0.05; **P < 0.01.

To elucidate the potential roles of EcRIP1 in fish virus replication, GS cells were transfected with pcDNA3.1-3HA or pcDNA3.1-EcRIP1 for 24 h and then infected with SGIV or RGNNV for a further 24 h. At the transcription level, the transcription levels of the SGIV major capsid protein (MCP), VP19, and ICP-18 genes, as well as the RGNNV CP and RdRp genes, were significantly inhibited when EcRIP1 was overexpressed (Figures 4A,B). These results indicated that EcRIP1 inhibited the replication of SGIV and RGNNV.

**Figure 5:**
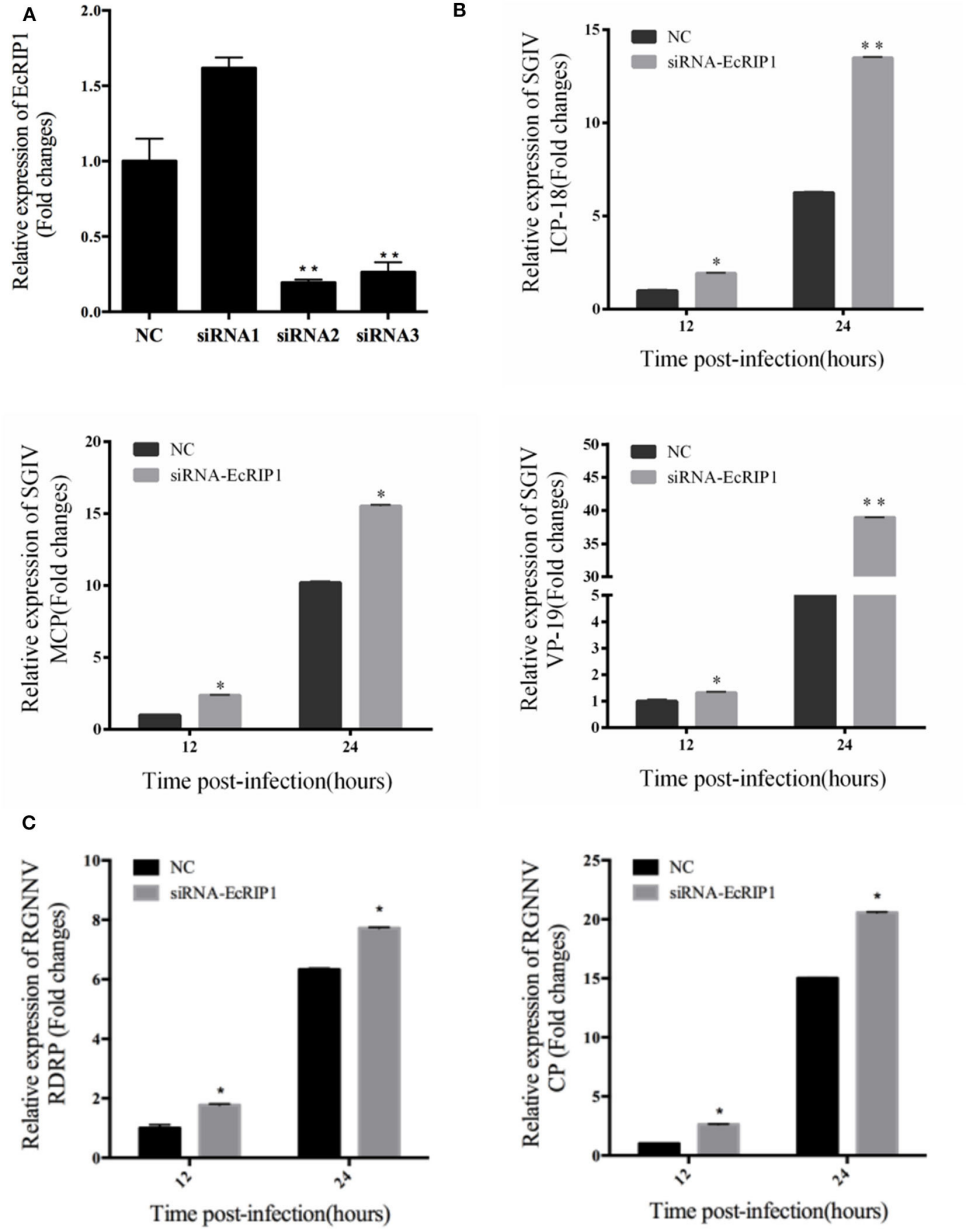
Knockdown of EcRIP1 by siRNA promoted SGIV and RGNNV replication *in vitro*. **(A)** GS cells were transfected with siRNAs targeting EcRIP1 or negative control siRNA (NC). At 48 h post-transfection, the RIP1 mRNA levels were determined by qPCR. **(B,C)** Viral gene transcription of SGIV or RGNNV in siRNA-EcRIP1- and NC-overexpressing cells. RIP1 knockdown and control cells were infected with SGIV **(B)** or RGNNV **(C)** for 24 h and collected at 24 h.p.i. to measure expression of ICP-18, MCP, and VP19 of SGIV and CP and RdRp of RGNNV (*n* = 3, mean ± SD). *P < 0.05; **P < 0.01.

To further investigate whether knockdown of EcRIP1 promotes SGIV or RGNNV replication, we designed three siRNAs targeting EcRIP1 and examined their interference efficiency in GS cells using qRT-PCR. Compared with the negative control siRNA, siRNA2 decreased expression of EcRIP1, with 74.5% knockdown efficiency (Figure 5A). After transfection with siRNA-EcRIP1 for 24 h, GS cells were infected with SGIV and RGNNV for a further 24 h, and then collected to examine the transcription of viral genes by qPCR. Knockdown of EcRIP1 by siRNA promoted SGIV and RGNNV replication compared with the cells transfected with the negative control siRNA (Figures 5B,C). These results suggested that EcRIP1 exerted antiviral effects on SGIV and RGNNV infection.

### EcRIP1 Overexpression Positively Regulated the Interferon Immune Response and Pro-inflammatory Cytokines

**Figure 6:**
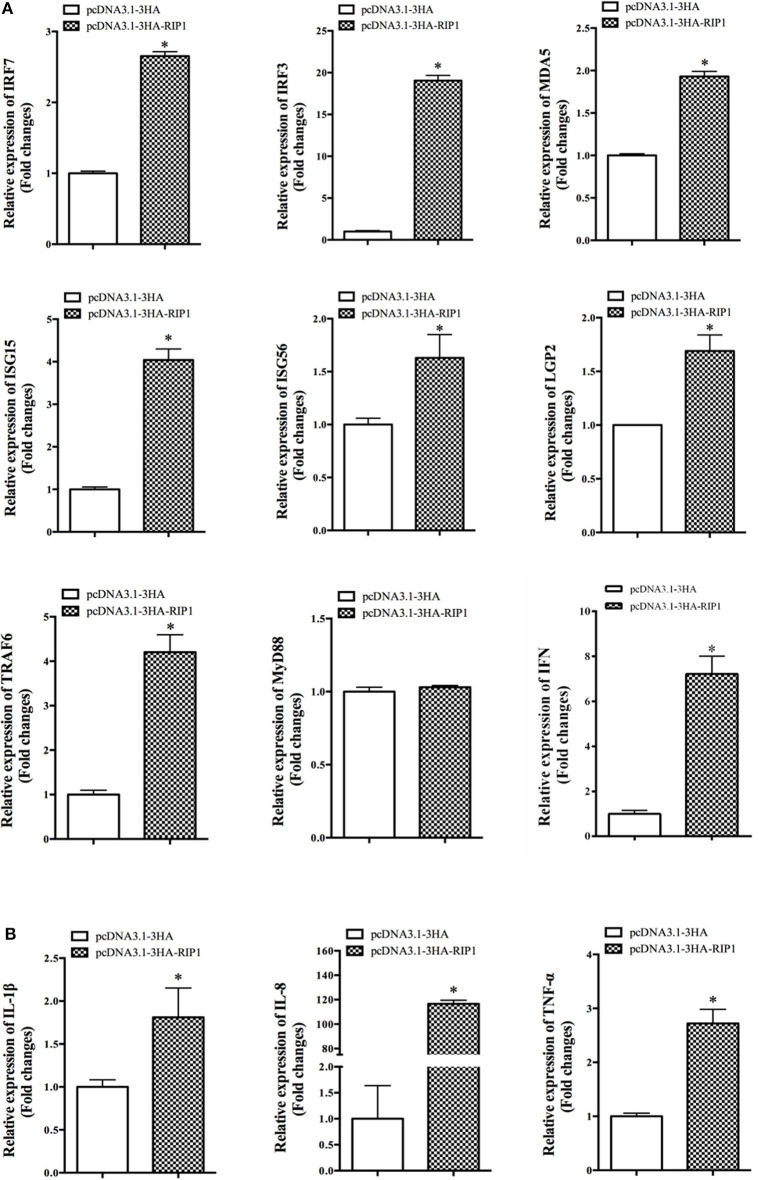
Overexpression of EcRIP1 positively regulated the IFN immune and inflammatory responses. GS cells were transfected with pcDNA3.1-3HA and pcDNA3.1-EcRIP1 and collected at 48 h to detect the expression of **(A)** IFN and IFN-stimulated genes including IRF3, IRF7, MDA5, ISG15, LGP2, ISG56, TRAF6, and MyD88 and **(B)** pro-inflammatory factors including IL-1β, IL-8, and TNF-α in pcDNA3.1-3HA- or pcDNA3.1-EcRIP1-overexpressing cells by qPCR (*n* = 3, mean ± SD). *P < 0.05.

To explore the potential mechanism involved in the action of EcRIP1 in fish virus infections, the roles of EcRIP1 on the host interferon immune and inflammation responses were evaluated by qRT-PCR. Compared with the controls, overexpression of EcRIP1 significantly increased the expression levels of IFN and IFN-stimulated genes, such as IRF3, IRF7, MDA5, ISG15, LGP2, ISG56, and TRAF6, but it had no effect on MyD88 (Figure 6A). In addition, the expression levels of pro-inflammatory cytokines such as interleukin (IL)-1β, IL-8, and TNF-α were all significantly increased in EcRIP1 overexpressing cells (Figure 6B).

### EcRIP1 May Interact With EcTRADD and Synergistically Upregulate SGIV-Induced Apoptosis

**Figure 7:**
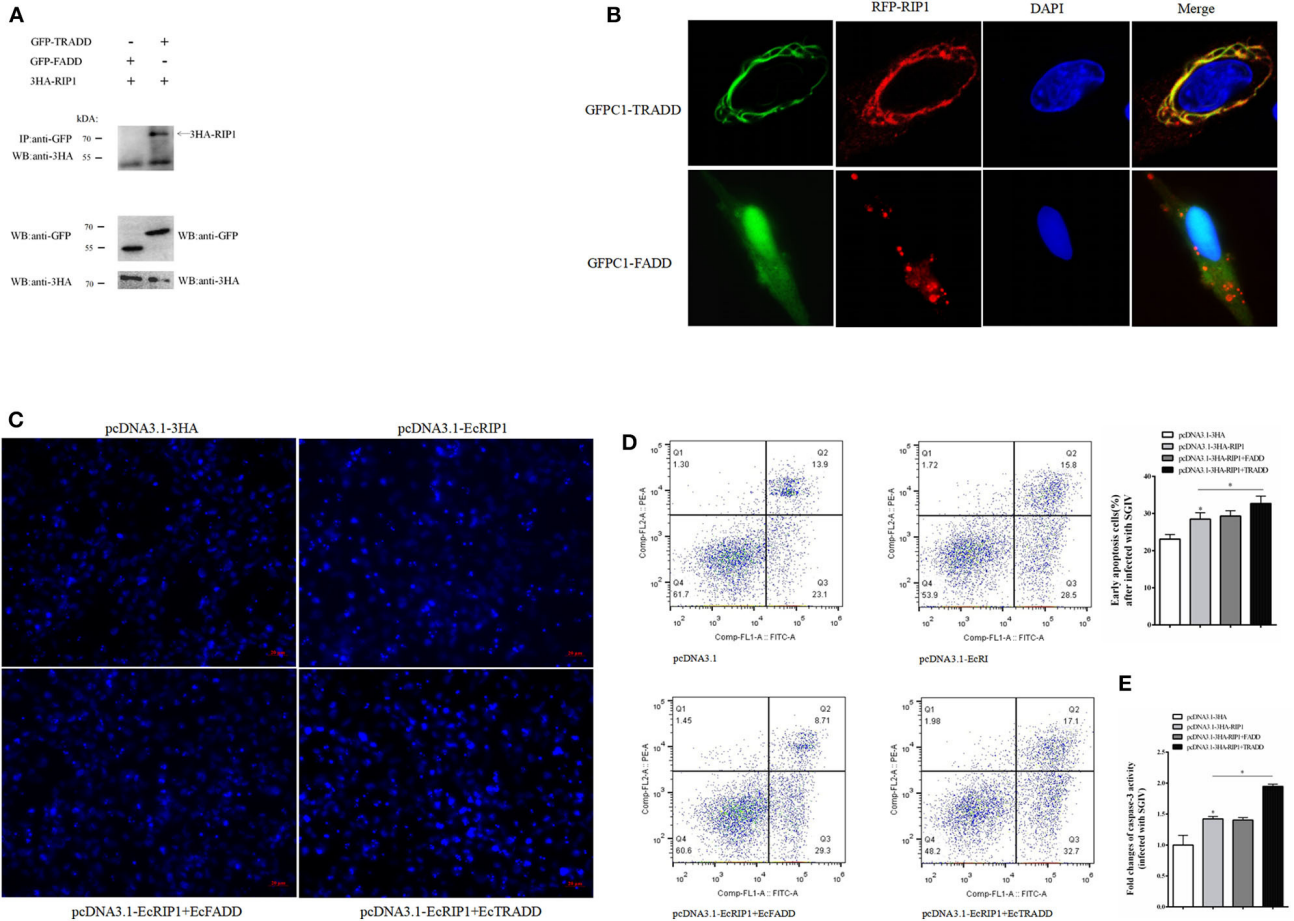
EcRIP1 may interact with EcTRADD and synergistically up-regulate SGIV-induced apoptosis. **(A)** Co-immunoprecipitation results in GS cells showing that EcRIP1 may interact with EcTRADD but not with EcFADD. Arrowheads indicate the specific bands. **(B)** GS cells were co-transfected with RFP-fused EcRIP1 with GFP-tagged EcFADD or EcTRADD for confocal laser scanning microscope analysis. Data are representative of at least three independent experiments in which 80% of the cells showed similar staining patterns. **(C–E)** Effect of EcRIP1 and EcTRADD on SGIV-induced apoptosis. **(C)** Cellular nuclear morphology in SGIV-infected cells; arrows indicate the apoptotic bodies. **(D)** Flow cytometry analysis of DNA content in SGIV-infected cells. Q3 represents the percentage of early apoptotic cells, and the differences between early apoptotic cells were analyzed. **(E)** The activities of caspase-3 in pcDNA3.1–EcRIP1, pcDNA3.1–EcFADD+EcRIP1, pcDNA3.1–EcTRADD+EcRIP1, and pcDNA3.1-overexpressed cells with SGIV infection. *P < 0.05.

We previously demonstrated that EcTRADD induced apoptosis in FHM cells infected with SGIV (30), whereas EcFADD inhibited it (24). Thus, we performed further analysis to assess the functional relevance of the interaction among EcTRADD, EcFADD, and EcRIP1, which all contain DD. The results of confocal assays showed that EcRIP1 accumulated in the form of dots on the cytoplasmic filaments surrounding the nucleus that were formed by EcTRADD, thus co-localizing with EcTRADD but not with EcFADD (Figure 7A). The results of co-immunoprecipitation experiments further illustrated that EcRIP1 may interact with EcTRADD but not with EcFADD (Figure 7B).

SGIV is known to induce typical apoptosis in FHM cells (18). Using flow cytometry and microscope observations, we quantitatively analyzed the change in the apoptotic rate of cells overexpressing EcRIP1, EcTRADD, and EcFADD after they were infected with SGIV (Figures 7C,D). Compared with the control, the percentage of early apoptosis in cells (Figure 7D-Q3) overexpressing EcRIP1 alone increased. The percentage further increased in cells overexpressing EcRIP1 and EcTRADD together, and more apoptotic bodies were observed. However, the percentage of early apoptosis and the number of apoptotic bodies in cells overexpressing both EcRIP1 and EcFADD did not change significantly compared to cells overexpressing EcRIP1 alone. The total rate of apoptosis in cells transfected with pcDNA3.1-EcRIP1, co-transfected with pcDNA3.1-EcRIP1+EcFADD or pcDNA3.1–EcRIP1+EcTRADD, and the control group were 28.5, 29.3, 32.7, and 23.1%, respectively.

Caspase-3 is a key mediator of apoptosis (32). To evaluate the possible involvement of downstream effector caspases, the activity of caspase-3 was detected after EcRIP1, EcTRADD, and EcFADD transfection. Caspase-3 activity was about 1.5 times higher in FHM/pcDNA3.1–EcRIP1 cells than in FHM/pcDNA3.1 cells and 1.4 times higher in FHM/pcDNA3.1–EcRIP1+EcTRADD cells than in FHM/pcDNA3.1–EcRIP1 cells 24 h after the addition of SGIV (*P* < 0.01) (Figure 7E). These results suggest that EcRIP1 and EcTRADD may interact to synergistically promote SGIV-induced apoptosis.

### EcRIP1 Participates in TRIF-Dependent but MyD88-Independent TLR Signaling

**Figure 8:**
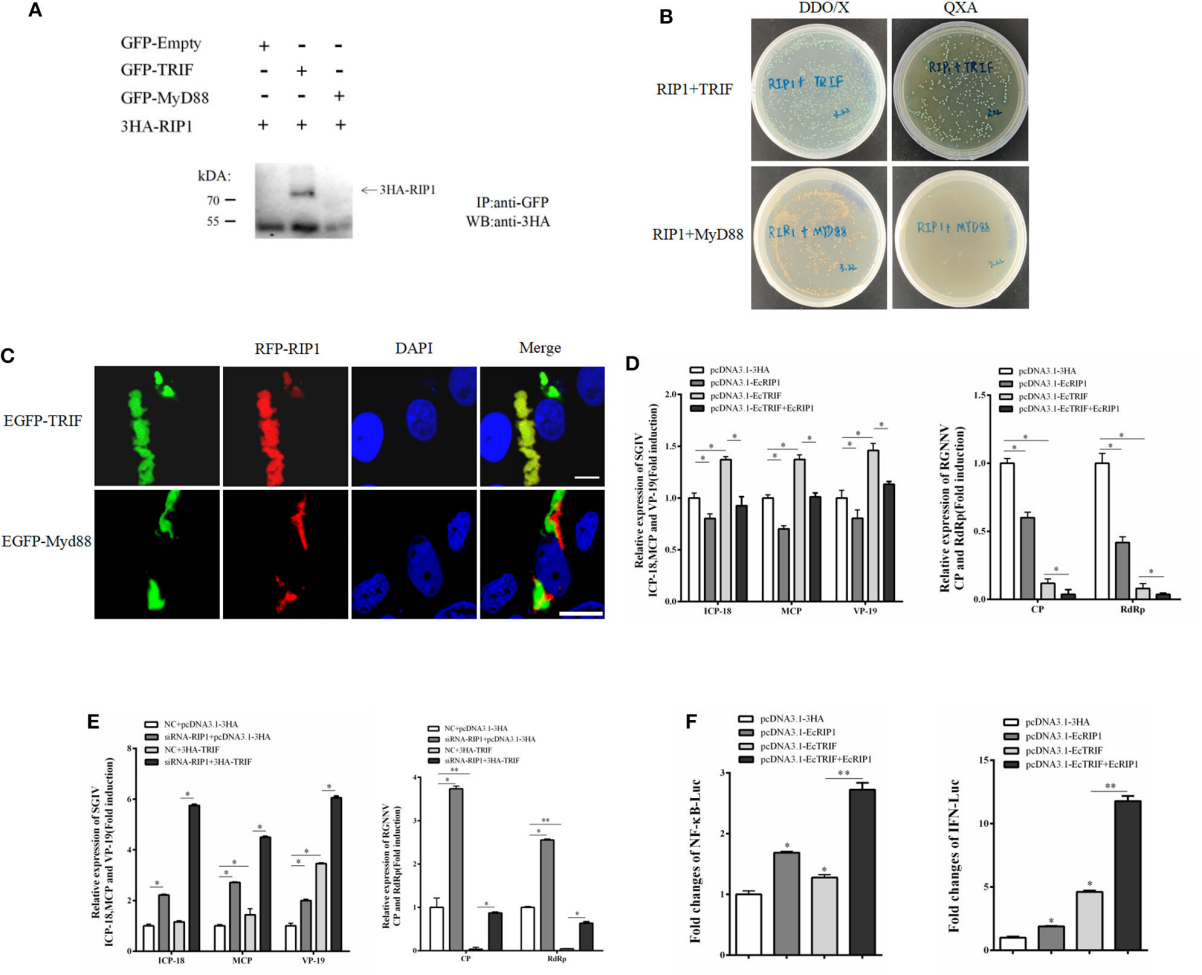
EcRIP1 participates in EcTRIF-dependent but EcMyD88-independent signaling pathways. **(A)** Co-immunoprecipitation results in GS cells showing that EcRIP1 may interact with EcTRIF but not with EcMyD88. Arrowheads indicate the specific bands. **(B)** For yeast two-hybrid analysis, pGBKT7-RIP1 and pGADT7-EcTRIF/EcMyD88 plasmids were constructed for interaction verification. The two plasmids were co-transformed into *S. cerevisiae* strain Y2H Gold. The transformants were tested on non-selective medium plate SD/-leu/-trp (DDO/X) to check whether the transformation is successful and the selective medium plate SD/-leu/-trp/-his/-ade/X-α-gal/AbA (QXA) to detect whether there is interaction between two proteins. **(C)** GS cells were co-transfected with RFP-fused EcRIP1 with GFP-tagged EcTRIF or EcMyD88 for confocal laser scanning microscope analysis. **(D)** Compared with cells transfected with pcDNA3.1-EcRIP1 or pcDNA3.1–EcTRIF alone, co-transfection with pcDNA3.1–EcTRIF and EcRIP1 significantly inhibited the transcription level of two genes of RGNNV, and reduced the promotion effect of EcTRIF on SGIV replication. **(E)** Compared with cells transfected with EcTRIF control alone, inhibition of EcRIP1 by siRNA can up-regulate the promotion effect of EcTRIF overexpression on SGIV virus replication and decrease the inhibition effect on RGNNV replication. **(F)** EcRIP1 and EcTRIF co-stimulated NF-κB and IFN activation. *P < 0.05; **P < 0.01.

In mammals, RIP1 participates in the MyD88-independent TLR3/4 pathway through association with TRIF. Based on our recent characterization of another TIR adaptor, EcTRIF (23), we further investigated whether EcRIP1 participates in the EcTRIF-mediated pathway. The results of confocal assays, yeast two-hybrid assays, and co-immunoprecipitation experiments showed that EcRIP1 may interact with EcTRIF but not with EcMyD88 (Figures 8A–C). Referring to previous studies (23), EcTRIF can significantly inhibit the replication of RGNNV virus, but promotes the replication of SGIV virus (Figure 8D). Compared with cells transfected with pcDNA3.1-EcRIP1 or pcDNA3.1-EcTRIF alone, co-transfection with pcDNA3.1-EcTRIF and EcRIP1 significantly inhibited the transcription level of two genes of RGNNV, and reduced the promotion effect of EcTRIF on SGIV replication (Figure 8D). We further verified the results by knocking down EcRIP1 with siRNA. Compared with cells transfected with EcTRIF control alone, inhibition of EcRIP1 by siRNA can up-regulate the promotion effect of EcTRIF overexpression on SGIV virus replication and decrease the inhibition effect on RGNNV replication (Figure 8E). EcTRIF was able to activate the promoter of NF-kB and the IFN response in GS cells, which is consistent with our previous research results. In addition, overexpression of EcTRIF and EcRIP1 together significantly upregulated the activation of NF-κB and IFN enhanced by EcTRIF alone (Figure 8F). These data suggest that EcRIP1 may interact with EcTRIF and participate in MyD88-independent TLR signaling.

### EcRIP1 May Interact With EcTRAFs

**Figure 9:**
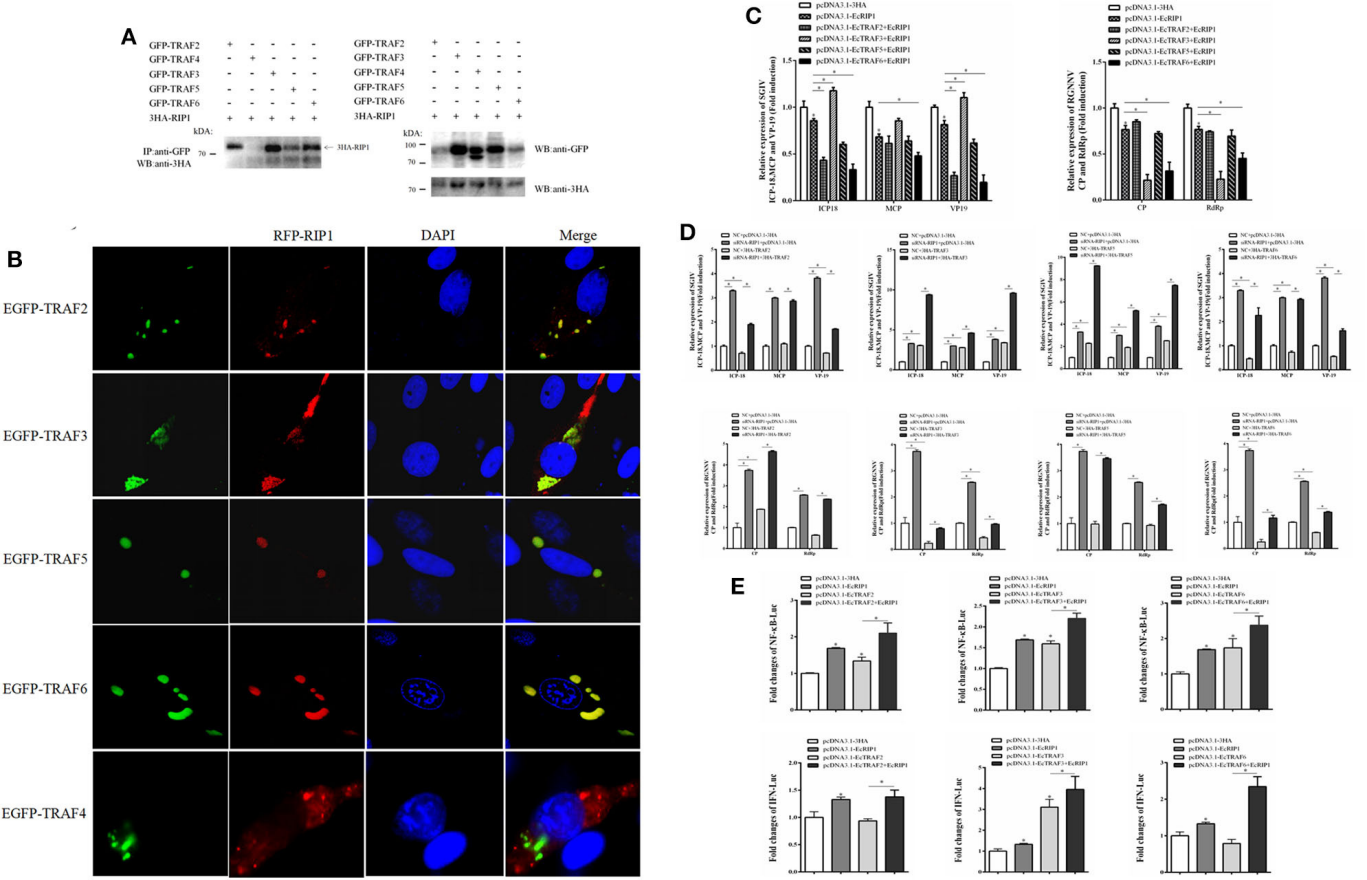
Effect of cooperation of EcTRAFs with EcRIP1 on NF-κB and IFN activation. **(A)** Co-immunoprecipitation results showed that EcRIP1 may interact with EcTRAF2, EcTRAF3, EcTRAF5, and EcTRAF6 but not with EcTRAF4 in GS cells. **(B)** Confocal laser scanning microscope images of GS cells co-transfected with RFP-fused EcRIP1 with GFP-tagged EcTRAFs. **(C)** EcTRAF6 significantly inhibited the transcription level of three genes of SGIV in synergy with EcRIP1. EcTRAF2 cooperated with EcRIP1 to inhibit transcription of ICP-18 and VP19 of SGIV, whereas EcTRAF3 promoted it. EcTRAF3 and EcTRAF6 cooperated with EcRIP1 to significantly inhibit the transcription levels of CP and RdRp belonging to RGNNV. **(D)** Compared with cells transfected with EcTRIF control alone, inhibition of EcRIP1 by siRNA can synergistically increased the promotion effects of EcTRAF3/5 on SGIV replication and reduced the inhibitory effects of EcTRAF2/6 on SGIV replication. Similarly, it also attenuates the inhibitory effects of EcTRAF3/6 on RGNNV replication. **(E)** The co-overexpression of EcTRAF2/3/6 and EcRIP1 significantly upregulated the activation of NF-κB and IFN regulated by EcTRAF2/3/6 alone. All reporter assays were performed in triplicate and repeated with three separate experiments. *P < 0.05.

EcRIP1 contains conserved TRAF2/3 binding sites (PXQXT/S) and TRAF6 binding sites (PXEXX), suggesting the potential ability to recruit TRAFs. The results of confocal assays and co-immunoprecipitation experiments showed that EcRIP1 may interact with EcTRAF2, 3, 5, and 6 but not with EcTRAF4. However, the results of the confocal assays showed that EcTRAF4 could change the original subcellular localization of EcRIP1 and transformed it into a cytoplasmic distribution (Figures 9A,B). EcTRAF6 significantly inhibited the transcription level of three genes of SGIV in synergy with EcRIP1 (Figure 9C). EcTRAF2 cooperated with EcRIP1 to inhibit transcription of ICP-18 and VP19 of SGIV, whereas EcTRAF3 promoted it. EcTRAF3 and EcTRAF6 cooperated with EcRIP1 to significantly inhibit transcription of CP and RdRp belonging to RGNNV, while EcTRAF2/5 had no significant effect on them (Figure 9C). We further verified the results by knocking down EcRIP1 with siRNA. Compared with cells transfected with EcTRAFs control alone, inhibition of EcRIP1 by siRNA can synergistically increased the promotion effects of EcTRAF3/5 on SGIV replication and reduced the inhibitory effects of EcTRAF2/6 on SGIV replication. Similarly, it also attenuates the inhibitory effects of EcTRAF3/6 on RGNNV replication (Figure 9D). Based on the effects of EcTRAF2/3/6 on the replication of SGIV or RGNNV, we performed further reporter gene assays to assess the functional relevance of EcTRAFs and EcRIP1. The co-overexpression of EcTRAF2/3/6 and EcRIP1 significantly up-regulated the activation of NF-κB and IFN regulated by EcTRAF2/3/6 alone (Figure 9E).

## Discussion

Numerous studies have shown that RIP serine/threonine kinase family members are essential sensors of cellular stress (4, 5). Among them, RIP1 is a key adaptor kinase for stress-induced signaling and a crucial regulator of cell survival and death. After exposure to different upstream signals, including viral infections, different and specific RIP1-containing complexes are formed, triggering different cellular responses (9). However, few studies of lower vertebrates have focused on how RIP1 acts as a key integrator of signaling pathways and exerts its antiviral function with interacting proteins in different signaling pathways. In this study, we cloned and characterized EcRIP1 and studied its interaction with specific proteins to explore the role of EcRIP1 in fish virus infection.

EcRIP1 encodes a 679 amino acid protein that shares 83.28% identity with *Perca flavescens* (XP_028449746.1). BLAST analysis indicated that in addition to sharing a homologous N-terminal kinase domain (S-TKc) with family members, EcRIP1 also bears a RIP isotype interaction motif (ID) and a C-terminal domain (DD) belonging to the superfamily of death domains, which allow recruitment to large protein complexes and initiation of different signaling pathways. Subcellular localization analysis showed that EcRIP1 was present in two forms (point-like uniform and dot-like aggregation around the nucleus), while several of its truncated mutants exhibited different subcellular localizations. Among them, the DD mutant of EcRIP1 showed a pattern of interconnected cytoplasmic filaments that was similar to the death effector filament (DEF) (31), while the ΔDD mutant showed cytoplasmic dispersion that did not form point aggregation. This result may indicate that DD plays an important role in the function of EcRIP1. This cytoplasmic filament that was present around the nucleus is consistent with that formed by EcTRADD, which is related to triggering apoptosis with nuclear fragmentation and inducing NF-κB activation (30, 33). In our study, the truncated mutants containing only the kinase domain of EcRIP1 did not activate the NF-κB response, whereas the truncated mutants containing the DD alone or together with the ID were more effective than the full-length EcRIP1. These findings indicated that activation of the NF-κB pathway is RIP1 kinase activity independent but DD or ID dependent.

RIP1 is essential for the antiviral immune response and participation in multiple signaling pathways (13, 14). Our qRT-PCR data showed that the concentration of EcRIP1 increased significantly during SGIV and RGNNV infection. Moreover, the transcription levels of viral genes were inhibited by EcRIP1 overexpression but enhanced by silencing of EcRIP1. In other words, EcRIP1 suppressed viral gene expression to inhibit infection of SGIV and RGNNV, which suggested that EcRIP1 plays a crucial role in the innate immune response against virus infection. To clarify the effects of EcRIP1 on the host IFN immune and inflammation responses, we examined the expression of IFN and IFN-stimulated genes and pro-inflammatory cytokines in EcRIP1-overexpressing cells. The expression levels of IFN and IFN-stimulated genes, such as IRF3, IRF7, MDA5, ISG15, LGP2, ISG56, and TRAF6 (but not MyD88), and inflammatory cytokines, such as TNF-α, IL-6, and IL-8, were all significantly increased. Thus, we speculate that EcRIP1 positively regulated IFN immune and pro-inflammatory responses to inhibit SGIV and RGNNV infection.

RIP1 can reportedly participate in the response to multiple cellular and antiviral pathways (9). Based on the inhibitory effect of EcRIP1 on SGIV and RGNNV, the interaction proteins that EcRIP1 may bind to and the antiviral signaling pathways that it may participate in were further studied to explore its antiviral mechanism. RIP1 can recruit TRADD through the carboxyl-terminal DD to regulate the TNF-R1-mediated apoptosis pathway (9, 10). Our results showed that EcRIP1 may interact with EcTRADD and co-localize with it in DEFs around the nucleus, but it could not interact with EcFADD.

It has been reported that all apoptosis conditions are related to the formation of DEFs (31) and that procaspases are effectively recruited into these structures (34). Results of previous experiments suggested that the DEF formed by EcTRADD may be related to nuclear fragmentation and formation of apoptotic bodies in the process of SGIV-induced apoptosis, thereby inducing SGIV-induced apoptosis through the recruitment and activation of procaspases (30). To evaluate this premise, nuclear staining and flow cytometry analysis were conducted, and caspase activities were measured in EcRIP1, EcTRADD+EcRIP1, and EcFADD+EcRIP1 overexpressed cells. Our data showed that overexpression of EcRIP1 promoted SGIV-induced apoptosis and increased the activity of caspase-3 in SGIV-infected overexpressing cells and that EcTRADD+EcRIP1 enhanced these effects. Therefore, we speculate that EcRIP1 may interact with EcTRADD and synergistically promote SGIV-induced apoptosis by forming cytoplasmic filaments.

Although studies have shown that RIP1 can participate in the antiviral pathway of RIG-like receptor signaling, the inhibitory pathway by which EcRIP1 acts against RGNNV virus is still unclear (12). In this study, we found that EcRIP1 may interact with EcTRIF and participate in MyD88-independent TLR signaling by up-regulating the activation of NF-κB and IFN. Because RGNNV is an RNA virus, EcRIP1 can inhibit the replication of RGNNV by targeting viral RNA by participating in the TLR3/TRIF-dependent signaling pathway. The result is consistent with the widespread antiviral effect of RIP1 on certain viruses that has been demonstrated in mammals (13, 14). TRIF-dependent signaling pathways also include TLR19 pathway in teleosts (35).

In humans, after DR stimulation, RIP1 and TRAF2 are recruited by TRADD to the DD of DRs to mainly activate NF-kB (36, 37). TRAF2 and TRAF5 are closely related members of the TRAF family of proteins, which are recruited and assembled with RIP1 into to the pathogen-induced TNF-α signaling pathway to activate NF-κB (38–40). Recently studies have shown that TRAF3 is an essential component of the TLR3-signaling pathway (41). TLR3 participates in type I IFN-β pathway via TRIF and TRAF3, which ultimately leads to inflammation and an immune response (42). Together with TRAF6, RIP1, and TRIF were reported to be recruited to participate in the TLR3/4 signaling involved in type I IFN-β pathway and to contribute to TRIF-induced NF-κB activation (11). In our previous study, overexpressed TRAF6 from *Epinephelus tauvina* significantly inhibited the transcription of SGIV genes in GS cells (22). Similar to human RIP1, our results showed that EcRIP1 may not only interact with EcTRAF2 but also with EcTRAF3, EcTRAF5, and EcTRAF6. EcTRAF6 significantly inhibited the transcription level of three genes of SGIV in synergy with EcRIP1. EcTRAF2 cooperated with EcRIP1 to inhibit transcription of ICP-18 and VP19 of SGIV, whereas EcTRAF3 promoted it. Additionally, EcTRAF3 and EcTRAF6 cooperated with EcRIP1 to significantly inhibit the transcription levels of CP and RdRp of RGNNV, whereas EcTRAF2/5 had no significant effect on them. Reporter gene assay results showed that EcTRAF2/3/6 and EcRIP1 significantly up-regulated the activation of NF-κB and IFN regulated by EcTRAF2/3/6 alone. Therefore, we speculate that EcRIP1 may form a complex with EcTRAF2 and EcTRADD to regulate the SGIV-induced TNF-α signaling pathway, thereby inhibiting SGIV replication. EcRIP1 also may participate in type I IFN through EcTRIF and EcTRAF3 in the TLR3/TLR19 pathway, culminating in inflammation and immune reactions against RGNNV. EcTRAF6 may be recruited by EcRIP1 to participate in activation of the TLR3/4 signaling pathway in response to SGIV or RGNNV.

In summary, EcRIP1 was cloned and characterized, and the responses of EcRIP1 to SGIV or RGNNV challenges were investigated. Intracellular localization was analyzed for EcRIP1 and its domains *in vitro*. Because EcRIP1 has a positive effect on the host IFN immune response and is associated with multiple molecules and thus targets multiple signaling pathways, overexpression of EcRIP1 *in vitro* can significantly inhibit the replication of SGIV and RGNNV. To our knowledge, it is the first time to report the roles of orange-spotted grouper RIP1 in the determination of cell fate under virus infection. Our findings will contribute to the understanding of the function of fish RIP1 in response to viruses.

## Data Availability Statement

The original contributions presented in the study are included in the article/supplementary material, further inquiries can be directed to the corresponding author/s.

## Ethics Statement

The animal study was reviewed and approved by The Animal Care and Use Committee of College of Marine Sciences, South China Agricultural University.

## Author Contributions

QQ and JW designed the experiments. XZ performed the majority of the experiments, analyzed the data, and wrote the manuscript. ZL, SW, and MS contributed the experimental suggestions. All authors revised the manuscript.

## Conflict of Interest

The authors declare that the research was conducted in the absence of any commercial or financial relationships that could be construed as a potential conflict of interest.
